# Cas9-expressing HC-04 hepatocytes facilitate CRISPR-based analysis of *Plasmodium falciparum* sporozoite-host interactions

**DOI:** 10.1371/journal.pgen.1012137

**Published:** 2026-05-18

**Authors:** Lisa H. Verzier, Eva Hesping, Marcel Doerflinger, Marco J. Herold, Justin A. Boddey

**Affiliations:** 1 The Walter and Eliza Hall Institute of Medical Research, Parkville, Victoria, Australia; 2 Department of Medical Biology, University of Melbourne, Parkville, Victoria, Australia; 3 Olivia Newton-John Cancer Research Institute, Heidelberg, Victoria, Australia; 4 School of Cancer Medicine, La Trobe University, Heidelberg, Victoria, Australia; University of Washington / Seattle Children’s, UNITED STATES OF AMERICA

## Abstract

Sporozoites of *Plasmodium falciparum*, the deadliest malaria parasite, are injected into the skin by infected mosquitoes and must reach the liver to initiate infection. There, they invade hepatocytes and develop into exoerythrocytic merozoites that eventually enter the bloodstream and invade erythrocytes, causing malaria. The sporozoite’s journey requires cell traversal, where sporozoites transiently enter and exit host cells, lysing membranes to move deeper into tissue and evade immune cell destruction. After reaching the liver and traversing several hepatocytes, sporozoites productively invade a final hepatocyte to establish an exoerythrocytic form. The molecular mechanisms underlying traversal, invasion, and intracellular development remain incompletely understood, particularly with respect to host factors. To address this, we engineered human HC-04 hepatocytes, the only known cell line supporting *P. falciparum* liver-stage development, to express Cas9-mCherry, enabling CRISPR-based functional genomics studies. We validated Cas9 activity of HC-04.2B3 and demonstrated successful guide-RNA-directed gene disruption via non-homologous end joining. Optimized traversal and invasion assays led to a robust cytometric readout suitable for screening human genes involved in *P. falciparum* infection. Disruption of 10 human genes previously implicated in infection by bacterial and viral pathogens confirmed utility of this platform. This study provides the basis for genome-wide CRISPR screens to uncover hepatocyte biology and host determinants of infection.

## Author summary

Malaria parasites first infect the liver before entering the blood and causing symptoms, yet studying this clinically silent stage in human cells has been technically challenging. As a result, few interactions between hepatocytes and parasites have been identified, limiting our capacity to target this early stage of infection with drugs or vaccines. Here, we developed a CRISPR-ready HC-04 hepatocyte line (clone 2B3) that stably expresses Cas9, enabling efficient host gene deletion in cells that support *Plasmodium falciparum* liver infection. We then optimized a harmonized assay that measures two early parasite behaviors in parallel: cell traversal, in which sporozoites wound and pass through hepatocytes, and invasion, defined as intracellular parasites quantified 24 hours post-infection. Successful disruption of 10 human genes validated Cas9-directed genome targeting and repair in HC-04.2B3 hepatocytes. While none of the targeted human genes was essential for *P. falciparum* sporozoite infectivity under the conditions tested, the generation of Cas9^+^ HC-04.2B3 establishes a robust and scalable platform for broader genetic screens to uncover host-pathogen interactions in *P. falciparum* pre-erythrocytic stages and potentially other hepatotropic pathogens.

## Introduction

Among the *Plasmodium* species responsible for human malaria, *P. falciparum* is the most prevalent, accounting for over 95% of cases and deaths globally [1]. Transmitted by the bite of an infected *Anopheles* female mosquito, *P. falciparum* sporozoites are deposited into human skin during blood feeding. To establish infection, sporozoites must migrate through the dermis and enter a blood vessel for transport via the bloodstream to the liver sinusoids. There, they migrate across the endothelium into the liver parenchyma, traverse hepatocytes before invading a final hepatocyte to continue their lifecycle [2–4]. *Plasmodium* sporozoites have evolved to interact with their host in multiple ways. First, through gliding motility that is characterized by start-and-stop movements due to successive attachment and cleavage of surface adhesins that enable migration through host tissues [3–5]. They have also evolved a remarkable mechanism called cell traversal, which allows them to cross physical barriers and avoid destruction following uptake by immune cells. Cell traversal refers to the sporozoites’ ability to pass through cells, entering and rapidly exiting them through the perforation and destabilization of host cell membranes that are rapidly repaired following the sporozoite breach *in vitro* [6–8]. Cell traversal is critical to infect mammalian hosts, as parasites deficient in a group of proteins related to the ‘membrane attack complex/perforin’ (MACPF) family are unable to efficiently traverse cells or infect the liver, even when delivered intravenously to bypass the dermal barriers [9–13]. Interestingly, even after reaching the liver, sporozoites traverse multiple hepatocytes before finally committing to productive invasion of a single hepatocyte. Parasites establish a parasitophorous vacuole (PV), where they mature into exoerythrocytic forms (EEFs) over the next 7 days before egressing and releasing merozoites, the stage that infects erythrocytes [6,14,15]. In contrast to productive invasion, traversing parasites briefly occupy a compartment called a transient vacuole (TV). Unlike the PV formed during productive invasion, the TV is marked by the presence of F-actin and phosphatidylinositol-3-phosphate (PI3P) and is rapidly lysed, whereas the PV lacks both proteins and remains intact [16,17] unless attacked by cell-autonomous innate responses that clear the infected cell [18].

Several pore-forming and MACPF-like proteins have been identified and are essential to the process of cell traversal. Sporozoite microneme protein essential for cell traversal (SPECT) and perforin-like protein 1 (PLP1, also called SPECT2) are pore-forming proteins that play key roles in cell traversal. Initially identified in rodent malaria models [9–12,19], both were later confirmed to be essential for *P. falciparum* cell traversal and sporozoite infectivity of humanized chimeric liver mice [12]. PLP1 has been shown to be essential during parasite egress from the TV, underlining its role in traversal [16]. Cell traversal protein for ookinetes and sporozoites (CelTOS) is another crucial factor for parasite motility and host membrane disruption and is involved in traversal by both ookinetes at the mosquito midgut and sporozoites in the mammalian host [20,21]. Biochemical studies revealed that CelTOS forms ~50 nm pores through preferential binding to phosphatidic acid, a lipid component enriched on the inner leaflet of host cell membranes. Its role in parasite exit from host cells has, however, yet to be conclusively demonstrated [22]. How these pore-forming proteins coordinate to disrupt host cell membranes, whether acting in concert or in sequence, remains unresolved. In addition, AMA1 and MAEBL, more commonly associated with invasion of erythrocytes and mosquito salivary glands have also been implicated in cell traversal and productive invasion of hepatocytes by *P. falciparum* sporozoites [23–25]. Despite the importance of cell traversal in establishing infection in the mammalian host, little is known about the host molecular mechanisms underlying this process. The overlap between parasite proteins involved in cell traversal and productive invasion suggests that these pathways may share common host-parasite interactions. Supporting this, several studies that did not differentiate between traversal and invasion have identified host cell processes, such as exocytosis and actin polymerization, as essential for sporozoite entry into hepatocytes by *Plasmodium* species that infect rodents [26,27]. Although the molecular determinants differentiating cell traversal from invasion remain incompletely defined, these findings imply convergence in the cellular machinery that may be exploited during both processes.

Host factors implicated in *Plasmodium* invasion differ across parasite and host species and model systems. CD81, the first host protein identified as essential for *P. falciparum* invasion of primary human hepatocytes [28,29] is also important for *P. yoelii* sporozoite invasion and rhoptry discharge in rodent models [30,31]. Other host receptors implicated in hepatocyte invasion or development were identified through genetic screening. Host factors involved in invasion and development of sporozoites were identified by short interfering RNA (siRNA) screens, identifying scavenger receptor class B type I (SR-BI) as important for *P. berghei*, and later by the human malaria parasite *P. vivax* [32–35], and PKCζ, involved in rodent malaria parasite growth [36]. Another approach involved comparing hepatocytes that were permissive, or not, to sporozoite infection to identify host proteins required for infection [35,37–39]. More recently, CRISPR approaches that offer robust and long-lasting gene disruption have been used to identify host factors for invasion and intracellular development of infectious pathogens, including apicomplexans such as *P. yoelii* [36,40–43]; however, CRISPR screens have not yet been reported with *P. falciparum* sporozoites. In *P. yoelii*, a CRISPR screen identified centromere protein J (CENPJ) as a negative regulator of rodent malaria parasite development in the liver [44]. This screen was performed in HepG2 human hepatocytes, which are useful for *Plasmodium* species that infect rodents but are not permissive to productive infection by *P. falciparum* sporozoites [45]. The human malaria parasite has been more challenging to study in *in vitro* culture, with a single cell line supporting liver infection [37,46,47]. While this model can sustain full liver-stage development and production of infectious merozoites, the invasion rates remain low [13,46,48–50] and HC-04 cells do not express appreciable levels of CD81 [48]*.* Additionally, anti-CD81 antibodies do not block invasion in this model, suggesting the parasite may exploit CD81-independent entry pathways.

Despite these limitations, HC-04 cells have been commonly used to investigate *P. falciparum* pre-erythrocytic biology [13,46,48–50], as they are more affordable than primary human hepatocytes and do not show donor to donor variations. Although the influence of experimental variables on *P. falciparum* HC-04 interactions has been explored [37,48], many aspects remain incompletely understood and the amenability of the cell line has yet to be fully leveraged. We therefore engineered Cas9-expressing HC-04 cells and validated clone 2B3 for CRISPR-based functional genomics studies in the context of *P. falciparum* liver-stage infection. Cas9^+^ HC-04.2B3 are a valuable cell line tool for genome-wide and arrayed CRISPR screens to uncover host determinants of liver-stage infection by human malaria parasites, including *P. falciparum* and *P. vivax*.

## Results

### Generation of Cas9^+^ HC-04 hepatocyte clones with endonuclease activity

**Fig 1:**
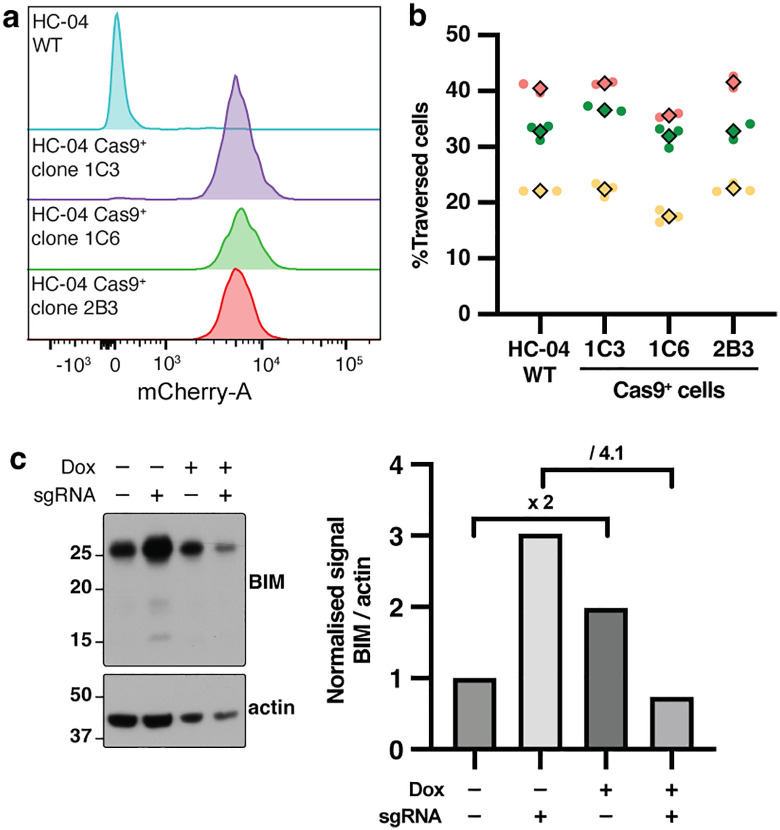
Generation of Cas9-mCherry^+^ HC-04 clones. **(a)** Cas9-mCherry^+^ HC-04 clones 1C3, 1C6 and 2B3 express mCherry^+^ as shown by flow cytometry. **(b)** Number of dextran^+^ (traversed) HC-04 cells. Only a modest reduction for clone 1C6 was observed (*p* = 0.0135, Dunnett multiple comparisons test). Colors represent biological replicates with both technical replicates (round points) and their average (diamond) shown. The multiplicity of infection (MOI) was MOI = 1 for green and red replicates and MOI = 0.5 for the yellow replicate. **(c)** Immunoblots show expression of BIM (~25 kDa) and actin (~45 kDa) in HC-04.2B3 transduced with sgRNA targeting *BIM* under expression of a doxycycline-inducible promoter (left). Densitometry of anti-human BIM and anti-human actin antibodies is shown (right) and the fold-change indicated numerically (above).

Given their prior use to investigate cell traversal and invasion by sporozoites [12,13,37,48–50], HC-04 hepatocytes were selected to enable functional studies of *P. falciparum*-hepatocyte interactions by genome editing. To this end, Cas9-expressing HC-04 cell line clones were generated by transduction of wild-type (WT) HC-04 cells with a lentiviral construct encoding Cas9 and the fluorescent protein mCherry separated by a 2A skip peptide (Cas9-mCherry). Successfully transduced cells were cloned by fluorescence-activated cell sorting (FACS) (**S1a Fig**) and single-cell clones were expanded. Three Cas9-mCherry^+^ clones, 1C3, 1C6, and 2B3, were identified by mCherry^+^ signal and selected for further characterization (**Fig 1a**).

To ensure that Cas9-mCherry expression did not affect *P. falciparum* sporozoite cell traversal, Cas9-mCherry^+^ HC-04 clones were assessed for traversal by sporozoites and compared to WT parental HC-04 cells by measuring their permissiveness to the uptake of dextran-FITC that is otherwise impermeable to HC-04 cells (gating strategy in **S1b Fig**) [12,13]. The percentage of traversed cells for WT HC-04 and all Cas9^+^ clones were similar (*p*>0.05). Clone 2B3 was selected for further characterization.

To confirm that Cas9 was functional when expressed in HC-04 cells, the endonuclease activity of clone 2B3 (hereafter called HC-04.2B3) was investigated following transduction of a doxycycline-inducible single guide RNA (sgRNA) targeting the human *BCL2L11* gene or a mock transduction lacking a sgRNA as a control [51]. The *BCL2L11* gene, also called *BIM*, was selected as reagents were readily available to study it, and sgRNA had been previously validated for potent knockdown efficiency [51]. Following 2 days of doxycycline treatment, HC-04.2B3 cells were analyzed by immunoblot for BIM protein expression. Doxycycline treatment increased BIM levels ~2-fold in the absence of sgRNA expression (**Fig 1c**), consistent with doxycycline-associated cellular stress [52,53]. In contrast, induction of the *BIM* sgRNA reduced BIM levels ~4.1-fold relative to the matched transduced population without doxycycline, corresponding to more than 75% reduction despite the doxycycline-associated upregulation observed in controls (**Fig 1c**), consistent with efficient Cas9-mediated disruption in a non-clonal population. The expression of endogenous BIM can be explained by either an incomplete transduction of HC-04.2B3 cells or a genome repair that was not deleterious to protein expression, or both.

Together, these results demonstrate that HC-04.2B3 expressed a functional Cas9 endonuclease able to effectively disrupt the *BIM* gene and mediate knockdown of BIM protein expression. This confirmed the suitability of HC-04.2B3 for broader genome editing applications to study interactions with *P. falciparum* sporozoites.

### Improving a *P. falciparum* cell traversal assay

To enable robust and high-throughput assessment of cell traversal by *P. falciparum* sporozoites, we assessed several parameters with HC-04.2B3 cells. In all cell traversal conditions tested, *P. falciparum* sporozoites dissected from mosquito salivary glands were incubated with hepatocytes for 4 hours to allow traversal to occur. This incubation time was based on our previous finding that cell traversal rates, measured as the percentage of dextran^+^ HC-04 cells, increase steadily during the first 2.5 hours post-infection (hpi) but plateaued thereafter [12].

**Fig 2:**
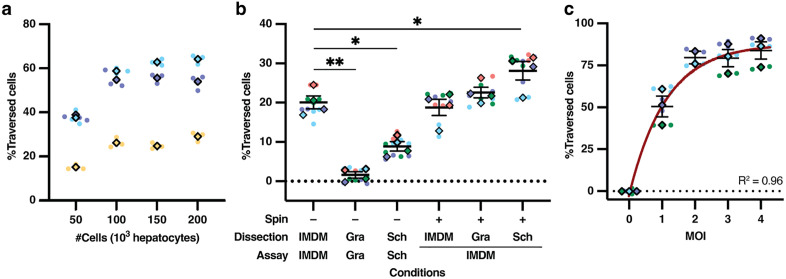
Cell traversal assay optimization with HC-04.2B3. **(a)** Seeding 100,000 cells led to improved cell traversal rates (however, not significant compared to 50,000 cells; Holm-Sídák multiple comparisons test). The MOI was 1 for blue and purple replicates and 0.5 for the yellow replicate. **(b)** Dissecting sporozoites into Schneider’s medium followed by centrifugation and resuspension in IMDM significantly improved cell traversal rates (Holm-Sídák multiple comparisons test). Different media for dissection and cell traversal assay (Dissection and Assay respectively) were tested, and a (spin +) included when they are different.**(c7 )** Increasing the MOI enhanced cell traversal rates, reaching a plateau at 88.4% at MOI 4 (brown curve; exponential plateau fit, *R²* = 0.96). Superplots [56] present each biological replicate (color), including technical replicates (round points) and their average (diamond). Mean ± SEM are shown in panels **b** and **c.** *p < 0.05, **p < 0.01, ***p < 0.001, ****p < 0.0001. Gra, Grace’s Insect medium; Sch, Schneider’s Insect medium.

First, we evaluated the influence of seeding density on cell traversal. HC-04.2B3 were seeded in 96-well plates at densities ranging from 50,000 cells (~80%, below confluence) to 100,000 cells (confluent monolayer) and higher densities that formed three-dimensional structures. Although differences were not statistically significant, seeding a confluent monolayer consistently increased cell traversal rates across three independent experiments and two multiplicities of infection (MOIs) (**Fig 2a**, *p* = 0.098). Next, we assessed how different media and centrifugation of sporozoites impacted viability and cell traversal activity. Dissection and incubation of sporozoites was performed using either Grace’s or Schneider’s insect medium or Iscove’s Modified Dulbecco’s Medium (IMDM). Insect media have been reported to improve sporozoite viability and extend their time of motility [54,55]. Dissecting sporozoites into Schneider’s medium, followed by centrifugation and resuspension in IMDM, yielded ideal cell traversal frequencies (**Fig 2b**, *p* = 0.036) while showing that centrifugation did not negatively affect sporozoites (**Fig 2b** *p* = 0.56). Finally, the MOI was increased from the standard 1:1 ratio (one sporozoite per hepatocyte [13]) to 4:1. This resulted in a higher proportion of dextran^+^ (i.e., traversed) cells. The increase was, however, non-linear and followed an exponential plateau, with a maximum cell traversal efficiency of 92.5% obtained at MOI 4 (**Fig 2c**, *R²* = 0.96).

### A refined and harmonized *P. falciparum* cell traversal and invasion assay

With optimized conditions for *P. falciparum* cell traversal established, we next assessed key parameters for quantifying sporozoite invasion, defined here as the frequency of hepatocytes harboring intracellular *Pf*CSP⁺ parasites. To enable higher-throughput quantification, we used flow cytometry following established workflows [13,23,24,39,50,57], including a study in which *Pf*CSP^+^ events were isolated and intracellular parasites were confirmed by microscopy [57]. Given prior findings and limited consensus in the literature (**Table 1**), we revisited three factors: sporozoite medium, MOI, and time for readout in order to evaluate the suitability of these variables for invasion assays, as detailed below.

**Table 1:** Different parameters for *P. falciparum* sporozoite invasion assays.

Invasion medium	MOI	Endpoint	References
*P. falciparum* invasion of HC-04 hepatocytes			
DMEM without glucose supplemented with 1 mM sodium pyruvate, 1% FBS, 200 units/mL penicillin, 200 mg/mL streptomycin, 1X MEM amino acids without L-glutamine and chemically defined lipid mixture	1	3-48hpi	[23]
	1	24hpi	[37]
DMEM/F12 supplemented with 10% HI-FBS, 15 mm HEPES, 1.5 g/L sodium bicarbonate, and pen/strep	1	24hpi	[48]
IMDM + 2.5% FBS supplemented with penicillin, streptomycin and L-glutamine	3	48hpi	[13]
DMEM/F-12 (Invitrogen), insulin 10 µg/mL (Sigma), 10% HI-FBS, 2% penicillin/streptomycin	0.3-0.5	3hpi	[58]
IMDM + 10% HI-FBS	0.625	3dpi	[59]
DMEM + 10% HI-FBS	0.25	2hpi	[60]
	0.3	1.5hpi	[39]
*P. falciparum* invasion of primary human hepatocytes			
InvitroGro HI Hepatocyte Media supplemented with Torpedo antibiotic mix	0.125	1.5hpi	[39]
	?	3dpi	[61]
William’s E medium supplemented with 10% HI-FBS, 2% penicillin- streptomycin, 1% sodium pyruvate, 1% l-glutamine, 1% insulin-transferrin-selenium	0.6	5dpi	[32]
	~1	3dpi	[25,28,31,62]
	~0.3	3-5dpi	[34,63]
	0.5	5dpi	[64]
William’s E medium with Glutamax supplemented with 10% heat-inactivated human serum, 1% insulin/transferrin/selenium, 1% sodium pyruvate, 1% MEM-NEAA, 1% Fungizone Antimycotic, 2% pen/strep and 1.6 µM dexamethasone	1	3dpi	[65]
F12 medium containing 10% HI-FBS, 0.2% bovine albumin, 10 pg/mL porcine insulin, and 5.10^-7^ M hydrocortisone hemisuccinate	?	3dpi	[33]
Dissection media: Schneider media Invasion media: InVitroGro CP Medium (Biorecla-mationIVT)	1	6dpi*	[54]
DMEM with high glucose, 10% (vol/vol) FBS, 0.5 U/ml insulin, 7 ng/mL glucagon, 7.5 μg/mL hydrocortisone and 1% (vol/vol) penicillin-streptomycin	3	3dpi, 6dpi	[66]
DMEM with high glucose, 10% FBS, 1% insulin-transferrin-selenous acid-linoleic acid, 7 ng/mL glucagon, 40 ng/mL dexamethasone, 15 mM HEPES, and 1% penicillin-streptomycin	~0.03, ~ 0.3	3hpi, 3dpi	[67]

hpi: hours post-infection, dpi: days post-infection, HI-FBS: heat-inactivated fetal bovine serum, MEM: minimum essential medium, NEAA: non-essential amino acids, *this assay measured development rather than invasion only.

**Fig 3:**
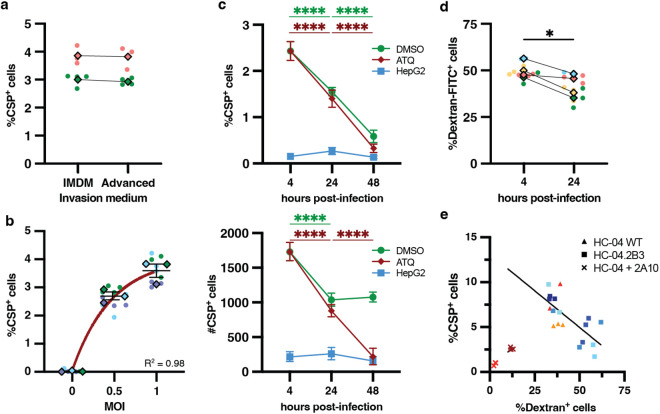
Harmonization of cell traversal and invasion assay parameters for *P. falciparum* sporozoites with HC-04 cells. **(a)** *Pf*CSP^+^ HC-04.2B3 cell quantification 4 hpi with *P. falciparum* sporozoites isolated in Schneider’s media prior to invasion assay in either IMDM or ‘Advanced’ medium. (*p* = 0.2, paired *t*-test). **(b)** Percentage of *Pf*CSP^+^ HC-04.2B3 cells increases with MOI in a non-linear fashion (paired *t*-test, exponential plateau, *R*^*2*^ = 0.98). **(c)** Percentage (left graph) and number (right graph) of *Pf*CSP^+^ HC-04.2B3 cells across different conditions: ATQ and DMSO are HC-04.2B3 cells treated with 50 nM atovaquone or 0.1% DMSO, respectively, from 4 hpi. HepG2 were used as a negative control as they are non-permissive to productive invasion by *P. falciparum* sporozoites. Tukey multiple comparisons test was used to check variation of each condition (colour) across time and significance was plotted. All conditions were performed at MOI = 0.5. **(d)** The percentage of traversed cells significantly decreases between 4h and 24 hpi (paired t-test), ranging from 4% up to 24% decrease. **(e)** Matched cell traversal and invasion rates using HC-04 WT (triangle), HC-04.2B3 (square) or HC-04 WT with inhibitory *Pf*CSP antibody 2A10 (cross). Cell traversal was quantified 4 hpi, invasion was quantified 24 hpi from the same wells. Color corresponds to biological replicates, line illustrates significant correlation (Pearson coefficient -0.978, *p*-value = 0.004). Superplots [56] in panels **a7, b** and **d** present each biological replicate (color), including technical replicates (round points) and their average (diamond).

Previous studies reported the improved invasion efficiency of HC-04 hepatocytes when *P. falciparum* sporozoites were dissected into the culture medium for mammalian cells, Medium 199 (M199), and then placed into ‘Advanced’ medium, comprising Dulbecco’s Modified Eagle’s Medium (DMEM) with supplements but lacking glucose (see methods), during the invasion assay [23,37]. Building on our findings regarding optimized cell traversal using insect medium for dissection, we then compared how invasion assay media (culture medium IMDM or ‘Advanced’) affected invasion rates when sporozoites were first dissected into Schneider’s insect medium (gating strategy in **S1c Fig**). As in the cell traversal assays, we quantified the number of HC-04.2B3 cells containing *P. falciparum* circumsporozoite protein-positive (*Pf*CSP^+^) parasites after 4 hours, capturing both productive invasion events and non-functional entry [23]. There was no significant difference in the percentage of HC-04.2B3 cells containing *Pf*CSP^+^ parasites between media conditions (**Fig 3a**; *p* > 0.05), indicating similar invasion rates when sporozoites were transferred from Schneider’s into either IMDM or ‘Advanced’ media. Therefore, subsequent assays involved sporozoite dissection into Schneider’s medium to preserve sporozoite viability, followed by placement into IMDM with serum and co-incubation with hepatocytes to allow sporozoite invasion.

Next, we assessed the effect of MOI on the rate of HC-04 invasion. Since cells that have been traversed are less likely to support infection [68], we focused on an MOI of 1 or lower, as higher MOIs caused more than half of the cells to be traversed (**Fig 2c**). As might be expected, incubation of *P. falciparum* sporozoites with HC-04.2B3 cells at MOI 1 resulted in significantly more *Pf*CSP^+^ host cells than MOI 0.5 (**Fig 3b**, *p* = 0.024). Interestingly, while MOI 0.5 consistently resulted in 2–3% *Pf*CSP^+^ HC-04.2B3 cells, doubling the number of sporozoites (MOI 1) did not result in twice the invasion rate measured at 4 hours (**Fig 3b**, exponential plateau, *R*^*2*^ = 0.98), suggesting that traversal of the majority of cells impacts invasion negatively (**Fig 2c**, 58% traversal at MOI 1). As such, an MOI 0.5 was selected for subsequent experiments.

Sporozoites either traverse host cells by generating and breaching a TV or invade the cell by forming a PV within which they develop into EEFs. Non-invading parasites are usually cleared within 12 hours [17]. With this in mind, we infected HC-04 cells with *P. falciparum* sporozoites and tracked the number of intracellular *Pf*CSP^+^ parasites for the first 48 hpi. As controls, HepG2 cells that are non-permissive to *P. falciparum* EEF development were included to determine non-functional entry events, as was the antimalarial liver-stage drug atovaquone (ATQ) to kill intracellular EEFs. In untreated controls, there was a reduction in both the number and percentage of *Pf*CSP^+^ cells between 4 and 24 hpi, consistent with clearance of some EEFs over time, as reported previously [23] which may be due to innate killing of parasites [18,69] (**Fig 3c**, *p* ≤ 0.0005). Interestingly, between 24 and 48 hours, *Pf*CSP^+^ counts were not significantly different (**Fig 3c**, bottom panel, *p* = 0.32); however, the proportion of *Pf*CSP^+^ cells decreased markedly due to host cell proliferation that diluted the proportion of infected cells (**Fig 3c**, top panel *p* < 0.0001). On the other hand, ATQ treatment caused a rapid clearance of infected *Pf*CSP^+^ HC-04 cells between 4h to 48h as expected [70], with the number of EEFs reaching the same background levels as those in non-permissive HepG2 cells (**Fig 3c**, Tukey multiple comparisons test *p* = 0.88). These results indicate that the 24 hpi timepoint was an appropriate proxy for distinguishing productive invasion from cell traversal events while mitigating the dilution effect of HC-04 replication evident by 48 hpi.

To study *P. falciparum* sporozoite interactions with hepatocytes, we reasoned it would be advantageous to develop a single assay allowing measurement of cell traversal and invasion rates from the same samples. To this end, we measured the cell traversal rates at 4 hpi by quantifying dextran^+^ cells in half of the experimental wells, while washing the remaining wells to remove extracellular sporozoites and incubating them under normal conditions overnight to allow intracellular parasites to continue their development before quantifying the number of *Pf*CSP^+^ and dextran^+^ cells again at 24 hpi. Assuming no significant difference in the number of dextran^+^ cells across time, this would allow the concomitant measurement of dextran^+^ (cell traversal) and *Pf*CSP^+^ (invaded) HC-04 cells at 24 hpi in the future. However, we observed a significant decrease in the proportion of dextran^+^ HC-04 cells at 24 hpi when compared to quantification made at 4 hpi, decreasing ranging from 4% up to 24% (**Fig 3d**, *p* = 0.034). This may be explained by HC-04 cell proliferation overnight diluting the dextran signal or by degradation of the fluorophore. We conclude that cell traversal is more accurately quantified on day 1 of the assay (4 hpi) while invasion rates are ideally quantified the following day (24 hpi). We then developed a harmonized quantitative assay that enables measurement of sporozoite traversal and invasion within the same infected cell population at 4 and 24 hpi (**Fig 3e**). Interestingly, an inverse correlation was observed between cell traversal and invasion (r = -0.71, *p* = 0.0009), while both mechanisms were effectively inhibited by addition of 10 µg/mL of *Pf*CSP antibody 2A10 as validated in individual assays [13,37,57]. This integrated design represents a methodological advance over existing approaches, which typically assess traversal and invasion in separate experimental setups, often under differing infection conditions. By capturing both parameters from the same sample, this assay provides a more accurate and internally controlled assessment of early sporozoite-hepatocyte interactions.

### Validation of HC-04.2B3 as a platform for host-pathogen interaction studies

**Fig 4:**
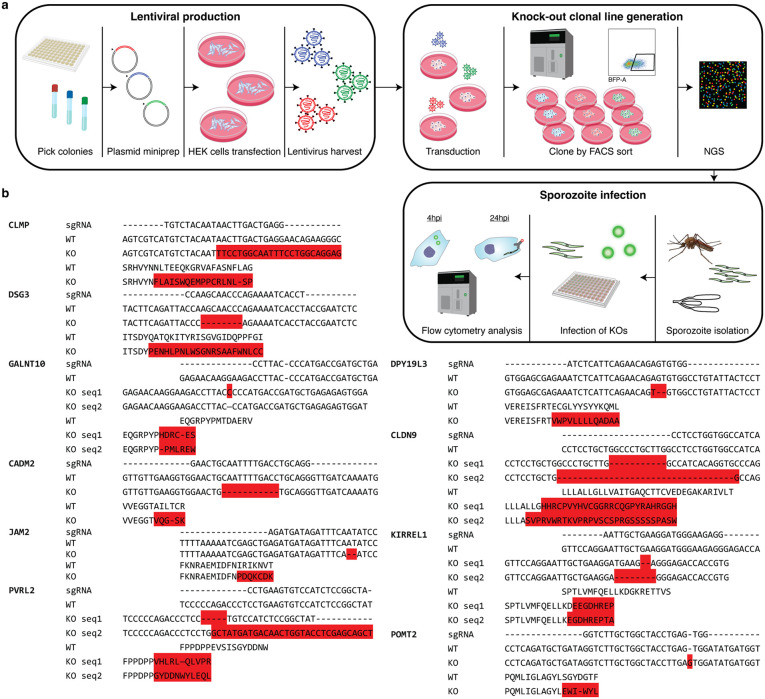
KO production and combined invasion and traversal assay workflow. **(a)** Selected host genes were knocked out in the HC-04.2B3 clone using sgRNA-expressing lentiviral particles. These were transduced into the target cells, followed by FACS sorting. Cas9-mediated cleavage introduced frameshift mutations in the genes of interest. Successful KOs were confirmed by NGS, and well-growing clonal lines were selected for functional validation in a combined traversal and invasion assay. Infected cells were assessed for dextran uptake at 4 hpi or CSP signal by immunofluorescence staining at 24 hpi. Cell schematics are by DBCLS (cell-MDCK, cell-CHO) and are licensed under CC-BY 4.0. All remaining icons are licensed under CC0 1.0 Universal, except for the sporozoites, salivary gland, antibody, and NGS icons, and the FACS plot, which are original work by the authors. **(b)** NGS confirmation of frameshift-inducing mutations in ten HC-04.2B3 KO clonal cell lines.

Having established HC-04.2B3 for the study of *P. falciparum* pre-erythrocytic infection, these cells were genetically modified to generate independent clones deficient in proteins involved in pathogen infection. Four receptors have been implicated in *Plasmodium* invasion, CD81, EphA2, SR-BI and AQP9 [28,31,32,38,39], though none could be a suitable positive control for validation. While AQP9 was identified after our selection [38], SR-BI is not directly involved in *P. falciparum* hepatocyte invasion [29,32], HC-04 invasion is CD81-independent [13], and the role of EphA2 is debated and likely related to parasite growth [39,71,72]. Thus, a subset of human genes encoding proteins with biological processes previously reported to be involved in pathogen entry into host cells and/or infection from other pathogens was selected for further study (**Table 2**). Genes were identified based on expression in the liver, location of their encoded proteins on the plasma membrane (or secreted beyond it), and glycosylation enzymes were also included in light of their importance for the conformation, function and surface trafficking of various substrates exploited by pathogens and the role of host proteoglycans in environmental liver sensing by *Plasmodium* sporozoites [37,73–75]. Initially, 19 human genes were chosen based on their known or suspected roles in pathogen infection, membrane dynamics, adhesion, endocytosis, and cytoskeletal regulation (**Table 2**). Of these, sgRNAs for 15 genes were present in a Sanger library available to our laboratory [76]. Each sgRNA plasmid was picked and amplified for lentiviral particle production. sgRNAs for all 15 genes were transduced into HC-04.2B3 in an arrayed format before cloning out with FACS of BFP fluorescence for genotyping and functional analyses of individual clones (**Fig 4a**). Knockout (KO) clones could not be generated for 5 genes (*RNF223, ESAM, NDST3, NDST1* and *CHIC2*) as the clones did not adhere normally or grew poorly in culture (**Table 2**), and these were not studied further. Clonal KO lines for the remaining 10 genes were successfully generated and validated by next generation sequencing (NGS) (**Figs 4b** and **S1**). Given the aneuploid nature of HC-04 cells, KO clones were considered validated if sequencing confirmed disruption of the sgRNA target site with wild-type sequences below 10% of the total sequences detected for each clone. Preference was given to clones harboring bi-allelic mutations (the one or two most dominant sequences) that introduced premature stop codons or frameshift mutations predicted to disrupt protein function.

**Table 2:** Selection criteria for forward screening.

Gene	Could be KO in HC-04	Present in the Sanger library	Expressed in liver	Plasma membrane or secreted	Cell-cell junction	Microbial infection	Involved in endo/exocytosis	Glycosylation	Significant in Vijayan screen [44]	Note
CADM2	✓	✓	✓	✓	✗	✓	✗	✗	✓	Involved in cell adhesion
CLDN9	✓	✓	✓	✓	✓	✓	✓	✗	✗	Cross-over endocytosis
PVRL2/NECTIN2	✓	✓	✓	✓	✓	✓	**~**	✗	✗	Recycled through endocytosis
CLMP	✓	✓	✓	✓	✓	✓	N/A	✗	✗	
JAM2	✓	✓	✓	✓	✓	✓	✗	✗	✗	Involved in *Salmonella Typhimurium* invasion
KIRREL1	✓	✓	✓	✓	✓	✗	✗	✗	✗	
DSG3	✓	✓	**~**	✓	✓	✗	✗	✗	✗	mRNA detected in the liver
GALNT10	✓	✓	✓	✓	✗	**~**	✗	✓	✓	MUC1 (product) is a receptor for *Salmonella*
POMT2	✓	✓	✓	**~**	✗	**~**	✗	✓	✗	Alpha-DAG1 (product) is viruses’ receptor
DPY19L3	✓	✓	✓	✗	✗	**~**	✗	✓	✗	Substrate interact with SaRS-CoV-2 & HIV proteins
ESAM	✗	✓	✓	✓	✓	**~**	✗	✗	✗	Involved in HCV pathogenicity
CHIC2	✗	✓	✓	✓	✗	✓	✓	✗	✗	Involved in vaccinia virus infection
NDST3	✗	✓	**~**	✗	✗	✓	✗	✓	✗	Involved in vaccinia virus infection.
NDST1	✗	✓	✓	✗	✗	✓	✗	✓	✗	Involved in chikungunya virus entry
RNF223	✗	✓	**~**	N/A	N/A	✓	✗	✗	✓	Involved in Sindbis virus entry
A4GALT	N/A	✗	✓	✗	✗	**~**	✗	✓	✓	Products are ligands for bacterial verotoxins
SEPT9	N/A	✗	✓	**~**	✓	✓	**~**	✗	✗	Localized to actin pole of invading bacteria
FBLIM1	N/A	✗	✓	✓	✓	✗	✗	✗	✗	Involved in integrin activation
GJC1	N/A	✗	✓	✓	✓	✗	✗	✗	✗	

Host candidates and their involvement in relevant pathways for *Plasmodium* cell traversal. ✓: gene is directly involved in the pathway, ~ : the relationship is indirect, ✗: signifies no relation, N/A: absence of conclusive data.

**Fig 5:**
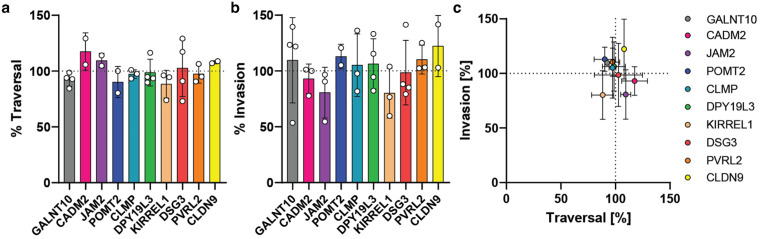
Inverse traversal and invasion rates of host KO candidates. Normalized (**a**) traversal and (**b**) invasion rates relative to respective control; no significant reduction was found for any of the lines (Kruskal-Wallis test with Dunn’s correction). Absolute traversal and invasion rates in **S1 Table**. **(c)** Invasion versus traversal rates, with 2-4 biological replicates per KO, mean ± SEM. Dashed line represents normalization to the HC-04.2B3 control.

To assess whether any of the 10 gene candidates are important for *P. falciparum* sporozoite infectivity, the rates of cell traversal and invasion were measured in the harmonized cytometric assay developed above (**Fig 4a**). Assays were performed in an arrayed format and compared to the parental HC-04.2B3 clone. Of the 10 candidates, none showed a significant decrease in either cell traversal or invasion by *P. falciparum* (**Fig 5a- 5c**). The absence of a significant effect on either mechanism suggests that the 10 genes selected are not involved in these processes, though functional redundancy as is the case in red blood cell invasion [77] could also explain the absence of a clear phenotype. All in all, the platform we generated, both the harmonized assay to measure cell traversal and invasion, and the Cas9^+^ HC-04.2B3 cell line, sets a robust foundation for systematic and scalable functional genomics approaches to uncover host determinants of *P. falciparum* liver-stage infection and other hepatotropic pathogens.

## Discussion

The molecular interplay between *P. falciparum* sporozoites and host hepatocytes is crucial to establish infection in humans yet incompletely understood. In this study, we engineered the tools necessary to begin dissecting the host determinants of early liver infection by the human malaria parasite at scale. By generating and validating HC-04.2B3 with functional Cas9-mCherry endonuclease activity, we enabled precise genetic manipulation of the only human cell line known to support *P. falciparum* liver-stage development. HC-04.2B3 demonstrated efficient Cas9 activity and retained normal susceptibility to sporozoite traversal and invasion, confirming the utility of these hepatocytes for functional studies with human malaria parasites.

We refined key experimental parameters to improve reproducibility and sensitivity of both cell traversal and invasion assays. Notably, decoupling mosquito dissection and assay media proved valuable: sporozoite traversal efficiency increased significantly when mosquitoes were dissected in insect medium rather than culture medium, consistent with prior reports on extended sporozoite viability in *P. vivax* [54,55]. This suggests that the benefit of ‘Advanced’ medium described previously may reflect improved sporozoite preservation rather than true enhancement of hepatocyte infection [37]. Additionally, the rates of cell traversal were more consistent when using confluent HC-04 monolayers, suggesting this may better mimic the physiological micro-environment. We also evaluated how sporozoite numbers influence the rate of cell traversal and invasion, with a plateau occurring at a MOI above 3 for the former. Interestingly, a small but consistent subset of cells (>5%) remained dextran^-^, raising the possibility that resistance to traversal by *P. falciparum* may naturally occur in a small proportion of the population.

On the other hand, the rate of HC-04 invasion by sporozoites plateaued at lower MOIs compared to cell traversal, perhaps due to cell traversal-induced changes to the cell that reduce cellular susceptibility, such as NF-κB activation that was reported previously [68]. This highlights the importance of carefully selecting the MOI in future genetic screens, not only to preserve the physiological relevance of the invasion process but also to ensure sensitivity of detection, in particular considering the limited availability of sporozoites. Temporal resolution also proved important for measuring parasite infectivity. While intracellular staining at 4 hpi captures both arrested and invaded parasites, this time point cannot clearly distinguish between the two events. By 24 hpi however, non-viable sporozoites are typically cleared [16,17], providing a clearer snapshot of productive invasion that occurred the day before, while limiting confounding effects from cell proliferation on the measure. Although sporozoites can abort traversal, lodging inside the nucleus and persisting for some time in HepG2 cells [28], these events are rare and we believe unlikely to affect the results measured in the harmonized cytometric assay we developed. As such, we conclude that 24 hpi represents a reliable endpoint for quantifying *P. falciparum* sporozoite invasion but is unsuitable for assessing the rate of cell traversal. We acknowledge that definitive discrimination between productive invasion and alternative intracellular uptake mechanisms would require either direct microscopy or a validated parasitophorous vacuole membrane marker in this assay context, though related flow-cytometric workflows have previously been supported by orthogonal microscopy in human hepatocytes [13,57]

Our harmonized assay, which allows concurrent measurement of cell traversal and invasion in the same sample at distinct timepoints, increases efficiency and reduces sporozoite usage by half. Importantly, traversal and invasion represent two distinct but intricately related events during the establishment of liver infection (**Fig 3e**, [68]). Sporozoites unable to traverse arrest inside host cells, leading to higher “invasion” rates at early timepoints which can lead to misleading conclusions [10,16,17]. Our harmonized approach resolves this limitation by linking traversal and invasion data from matched samples. Interestingly, we observed an inverse correlation between traversal and invasion events. This could reflect an early shift in sporozoite behavior from traversal to invasion [2,3] i.e., lower traversal coinciding with higher invasion rates as parasites spend less time migrating through cells and more time locating a permissive hepatocyte in which to establish a parasitophorous vacuole. A host-centered hypothesis could also account for this pattern: traversal-associated wounding promotes release of cytosolic material into the medium, thereby enhancing NF-κB activation, innate immune responses, and ultimately parasite clearance [68]. These explanations are not mutually exclusive, and a combination of parasite- and host-driven effects may underlie the inverse correlation. Resolving their relative contributions will require targeted follow-up studies. This assay provides a robust and scalable platform to dissect early host-parasite interactions, assess the functional impact of genetic perturbations in either host or parasite, and evaluate pharmacological interventions that may differentially affect traversal versus invasion. In doing so, it offers a more holistic and physiologically relevant framework for studying the early establishment of *Plasmodium* liver stage and should be investigated simultaneously when studying this stage of the parasite life cycle.

Applying these conditions and assay, we validated both protocol and cell line suitability by targeting a small number of human genes exploited by pathogens for a role in *P. falciparum* sporozoite infectivity. None of the genes disrupted in this study were critical for *P. falciparum* cell traversal or invasion using the assays we developed, though they may be involved in downstream pathways essential for EEF development that our short-term assay does not capture. Additionally, specific characteristics of HC-04 cells might impact the results, as differences in productive invasion between primary human hepatocytes and HC-04 cells have been reported [13,28,29]. To our knowledge, a validated positive control host gene KO that reproducibly reduces *P. falciparum* traversal or invasion in HC-04 cells has not been established. Previous studies have reported that blocking CD81, a known entry receptor in primary human hepatocytes, does not significantly reduce sporozoite invasion in HC-04 cells [13], while a study on HC-04.J7 derivatives showed many previously implicated surface receptors were not detected or functional [37]. Thus, the screening of selected host genes in this study served primarily as a proof-of-concept application of the assay rather than an exhaustive validation of host factors involved in traversal or invasion. The absence of strong phenotypic effects likely reflects that the disrupted genes are not directly required for the early events captured in this short-term assay rather than limitations in assay sensitivity. However, we acknowledge that the restricted size of the candidate gene set and potential functional redundancy among host pathways represent limitations of this proof-of-concept study. Broader genome-wide or combinatorial perturbation approaches will be important to more comprehensively uncover host determinants of traversal and early infection to fully leverage this platform.

Future work will extend the assay to capture later stages of liver-stage development by maintaining infected cultures for longer periods and transferring them into larger wells to prevent overcrowding, as well as incorporating additional readouts such as early EEF size, parasite maturation, and host cell responses to further refine this system. In addition, the inclusion of positive host genetic controls, particularly perturbations of pathways previously implicated in liver-stage development, such as autophagy-related components, will further validate the platform’s capacity to detect biologically meaningful effects on intracellular parasite growth. Together, these extensions will position the system as a robust tool for dissecting early post-entry host-parasite interactions governing parasite establishment and development.

Together, our study underscores the value of engineered HC-04 cell lines and refined, harmonized phenotypic assays as foundational tools for human liver-stage malaria research and for translational studies of other hepatotropic pathogens. Future efforts should aim to extend this platform to longer-term assays for EEF development, combinatorial gene KOs, or larger pooled CRISPR screens to uncover the complex network of host factors influencing *P. falciparum* liver-stage biology.

## Materials and methods

### Cell maintenance and lentivirus production

HC-04 hepatocytes [46] were routinely cultured in Iscove’s Modified Dulbecco’s Medium (IMDM, Life Technologies Cat #12200) supplemented with 5% fetal bovine serum (FBS) and penicillin and streptomycin (pen/strep). HEK293T cells were maintained in Dulbecco’s Modified Eagle Medium (DMEM, Life Technologies Cat #31600) complemented with 10% FBS and pen/strep. Both cell lines were passaged every 2–4 days and cultured for no more than 2 months.

### Lentiviral production, transduction and virus titration

Lentivirus production was performed as previously described [78]. Briefly, HEK293T were seeded 24h prior to calcium phosphate co-transfection of target DNA with envelope, packaging, replication plasmids (from Didier Trono, Addgene #12251, Addgene #12253 and Addgene #12259 respectively, [79]). For Cas9, FUCas9Cherry plasmid was used as target DNA (Addgene #70182, [51]). For the individual gene KO, gene-specific sgRNAs (N = 2) derived from the Sanger library were used as target DNA [76]. Cells were incubated overnight, and medium was changed the following morning. Both 24h and 48h supernatants were collected, filtered through 0.45 µm and stored at -80ºC until further use.

HC-04 cells were seeded at 100,000 cells per well in 6-well plates the day prior to transduction. For Cas9 transduction, 5 mL of lentiviral supernatant containing 2 µg/mL of polybrene were added and particles were centrifuged onto cells at 2,200 rpm for 2h at 35ºC. Cells were washed twice and incubated 24h before a second transduction was performed. Similarly, sgRNA transduction of individual genes were performed by a single transduction using 2 mL of lentiviral supernatant containing 2 µg/mL of polybrene. Cells were washed twice daily for the first 2 days. In all cases, transduction was confirmed two days later by detecting fluorescence on a BD LSRFortessa X-20 flow cytometer. Clonal cell lines were established by sorting individual fluorescent cells using a BD FACSAria flow cytometer, Cas9 clones being mCherry-fluorescent and target gene KO cell lines BFP-positive.

### Cas9 activity by immunoblotting

Cas9 activity was confirmed by immunoblotting. HC-04 Cas9^+^ 2B3 were transduced with the *isgBim* huEx3 construct [51] targeting the *BIM* gene with a doxycycline-inducible promoter or an empty particle. Two days post-transduction, transduced cells were split into two populations, one which was incubated with 1 µg/mL of doxycycline while the control stayed in culture medium for another two days. Cells were then trypsinized, washed twice with DPBS and dry pellets were frozen at -80°C. Immunoblotting was performed as previously [51] with minor adjustments. Briefly, cells were lysed in RIPA buffer containing 1X protease and phosphatase inhibitors for 30 min on ice. DNA material was pelleted at 10,000 x g for 5 min at 4°C. Total protein extracts were denatured at 96°C for 5 min and were separated on SDS-PAGE gel 4–12% polyacrylamide (Invitrogen NP0321). After transfer onto a 0.45 μm nitrocellulose membrane (Amersham), samples were blocked and probed (anti-BIM, Enzo Life Sciences ADI-AAP-330-E and anti-β-actin clone AC74, Sigma-Aldrich kindly provided by Prof Gemma Kelly, WEHI; anti-rabbit and anti-mouse IgG-HRP, Invitrogen) in 5% milk/Dulbecco’s phosphate buffered saline. Imaging was performed with either ECL Western Blotting Detection Reagent kit or the ECL Prime kit (Amersham) depending on signal. Super RX x-ray films (Fuji Film) were developed by Kodak X-OMAT processor.

### *P. falciparum* maintenance and gametocyte induction

*P. falciparum* NF54 was cultured in O-positive human blood (obtained from Melbourne Red Cross) as described [12]. Early parasites were synchronized using regular sorbitol treatment. Synchronous culture was used for gametocyte induction which was performed following the crash method [80], with daily medium changes for 16 consecutive days.

### Mosquito rearing and infection

*Anopheles stephensi* mosquitoes were infected with *P. falciparum* gametocytes (0.3% stage V gametocytemia, 50% hematocrit) 1–3 days post-eclosion. They were deprived of sugar for 48h to ensure only the fittest engorged females were conserved for subsequent dissection. Oocyst counts were performed 7 days post-infection using mercurochrome staining. Sporozoite dissections took place 14–18 days post-infection.

### Cell traversal assay

HC-04 cells were passaged, and 100,000 cells (or specified number) were seeded per well of a 96-well plate the day before the assay. Salivary gland dissections were carried out in Schneider’s insect medium (unless otherwise stated) and lasted no more than 1h. Sporozoites were released and filtered through glass wool before counting. They were pelleted for 3 min at 10,000 x g at 4ºC and resuspended in IMDM containing 10% heat-inactivated human serum (HIHS), 0.5 mg/mL dextran-FITC (Invitrogen D1821). After addition to cells, parasites were pelleted onto hepatocytes at 100 x g for 3 min and incubated for 4h at 37ºC. Cell traversal rates were analyzed by flow cytometry (BD LSRFortessa X-20 flow cytometer) after cells were trypsinized, washed and resuspended in DPBS. Dextran-FITC^+^ gates were set using matched no-sporozoite control wells, as shown in **S1b Fig**. Cell traversal rates were calculated by subtracting the percentage of dextran-FITC^+^ cells in these negative-control wells from that measured in wells containing sporozoites, thereby accounting for background uptake through alternative host-cell pathways such as endocytosis.

### Invasion assay

One day prior to the assay, 100,000 hepatocytes were seeded per well in a 96-well plate. Dissection and sporozoite isolation were performed as described above, and they were resuspended in IMDM or ‘Advanced’ medium (DMEM without glucose (Life Technologies, 11966-025) containing 1% active FBS (Corning, 35-010-CV), 1 mM sodium pyruvate (Life Technologies, 11360-070), 1X MEM non-essential amino acids without L-glutamine (Sigma-Aldrich, M5550), 1:500 dilution of Lipid Mixture 1, Chemically Defined (Sigma-Aldrich, L0-288) and 1X Pen/Strep (Corning, 30-001-Cl) [37]) supplemented with 10% HIHS for an MOI of 0.5. Plates were centrifuged at 100 x g for 3 min and incubated for 4h. Cells were washed once in DPBS and, for assays beyond 4h, cultured in IMDM supplemented with 5% FBS, 2.5 µg/mL amphotericin B, 110 µM neomycin and 50 µg/mL gentamicin. At the designated endpoint, cells were trypsinized, fixed and stained using Cytoperm kit (BD Biosciences, 554714) as previously described [39]. In short, fixation was performed in Cytofix for 15 min on ice, followed by two washes and staining in 1 X Cytoperm for 1 hour with 1 µg/mL of α-*Pf*CSP 2A10 monoclonal antibody (MR4, MRA-183A) conjugated to Alexa Fluor 647. Cells were washed twice in Cytoperm solution before being resuspended in DPBS and percentage of intracellular parasites was measured by flow cytometry (BD LSRFortessa X-20 flow cytometer). For calculation of the total number of intracellular parasites, half (50 µL) of the samples were analyzed. The total number of cells was initially calculated by adding the singlets and doublets (defined as the difference between total number of hepatocytes and singlets) and multiplying the sum by two. The absolute number of intracellular parasites was calculated as the product of the total number of cells analyzed and the percentage of PfCSP⁺ cells.

### Harmonized cell traversal and invasion assay

Harmonized assays were performed as cell traversal with the following adjustments. After 4h incubation at 37ºC, cells were washed with DPBS and trypsinized for 5 min. Wells were resuspended in 200 µL of IMDM supplemented with 5% FBS, 2.5 µg/mL amphotericin B, 110 µM neomycin and 50 µg/mL gentamicin. Half (100 µL) of the cell suspension was transferred into a 96-well round bottom and centrifugated at 300 x g for 3 min, before resuspension in DPBS and cell traversal assessment by flow cytometry (BD LSRFortessa X-20 flow cytometer). The remaining half (100 µL per well) was replated into a fresh 96-well flat bottom plate to prevent contamination rising from the mosquito debris added to the cells and incubated at 37ºC for another 20h after which hepatocyte invasion was determined as described above.

### Next Generation Sequencing

Genetic disruption of target genes was confirmed by next generation sequencing following overhang-based PCR preparation. Clonal cell lines were generated, and gene-specific primers were designed to flank target regions, producing 250–300 bp amplicons (300-cycle kit). Each primer included a universal 5′ sequencing overhang: Forward 5′-GTGACCTATGAACTCAGGAGTC-3′; Reverse 5′-CTGAGACTTGCACATCGCAGC-3′. PCR was performed in triplicate in 96-well format with a no-template control per primer pair. Each 20 µL reaction contained 1 µL genomic DNA (~100 ng/µL), 10 µL GoTaq Green Mix, 0.5 µL of each primer (10 µM), and 8 µL H₂O. Cycling conditions: 95 °C for 3 min; 18 cycles of 95 °C for 15 s, 60 °C for 30 s, 72 °C for 30 s; final extension at 72 °C for 7 min; hold at 10 °C. PCR products were cleaned using 20 µL (1:1) NGS beads, incubated 5 min, and separated on a magnetic rack. Supernatant was removed, and wells were washed twice with 150 µL 70% ethanol. After air drying, DNA was eluted in 40 µL H₂O, and 30 µL transferred to a new 96-well plate.

For indexing PCR, uniquely barcoded overhang primers (10 µM) were arranged so each clone received a unique primer pair. Each 20 µL reaction included 10 µL cleaned DNA, 10 µL GoTaq Green Mix, and 0.5 µL of each indexing primer. Cycling: 95 °C for 3 min; 24 cycles of 95 °C for 15 s, 60 °C for 30 s, 72 °C for 30 s; final extension at 72 °C for 7 min; hold at 10 °C. Reactions were spot-checked via agarose gel electrophoresis. For pooling, 5 µL from each well (including controls) were combined, mixed in a 25 mL reservoir, and transferred to a 1.5 mL tube. From this pool, 50 µL underwent bead cleanup using 40 µL NGS beads. After 5 min, beads were collected, washed twice with 180 µL ethanol, air-dried, and eluted in 105 µL H₂O. After 5 min, beads were cleared on a magnetic rack, and the eluate was used for sequencing. Libraries were sequenced on an Illumina MiSeq, and data were analyzed in DNASTAR MegAlign Pro by aligning reads to the HC-04.2B3 parent clone and respective sgRNA to confirm gene disruptions.

## Supporting information

S1 Fig Flow cytometry strategies. **(a)** HC-04 cells transduced with Cas9-mCherry construct were analyzed by flow cytometry before FACS cloning. **(b)** Cell traversal was determined by flow cytometry of live HC-04 4 hours post addition of sporozoites. Cells were gated before singlets were selected and dextran-FITC was determined using a well containing dextran-FITC in the medium but no sporozoites (top). **(c)** Similar strategy was used to determine invasion, though fixed cells (left) show different scatter profile.
(TIF)

S1 Table Numerical data for all graphs, including absolute traversal and invasion rates of assays normalized as shown in Fig 5. (XLSX)
