# Supplementary figures and images for: Cas9-expressing HC-04 hepatocytes facilitate CRISPR-based analysis of Plasmodium falciparum sporozoite-host interactions
Source: PLoS Genet. 2026 May 18;22(5):e1012137. doi: 10.1371/journal.pgen.1012137 (PMC13215610; doi:10.1371/journal.pgen.1012137)

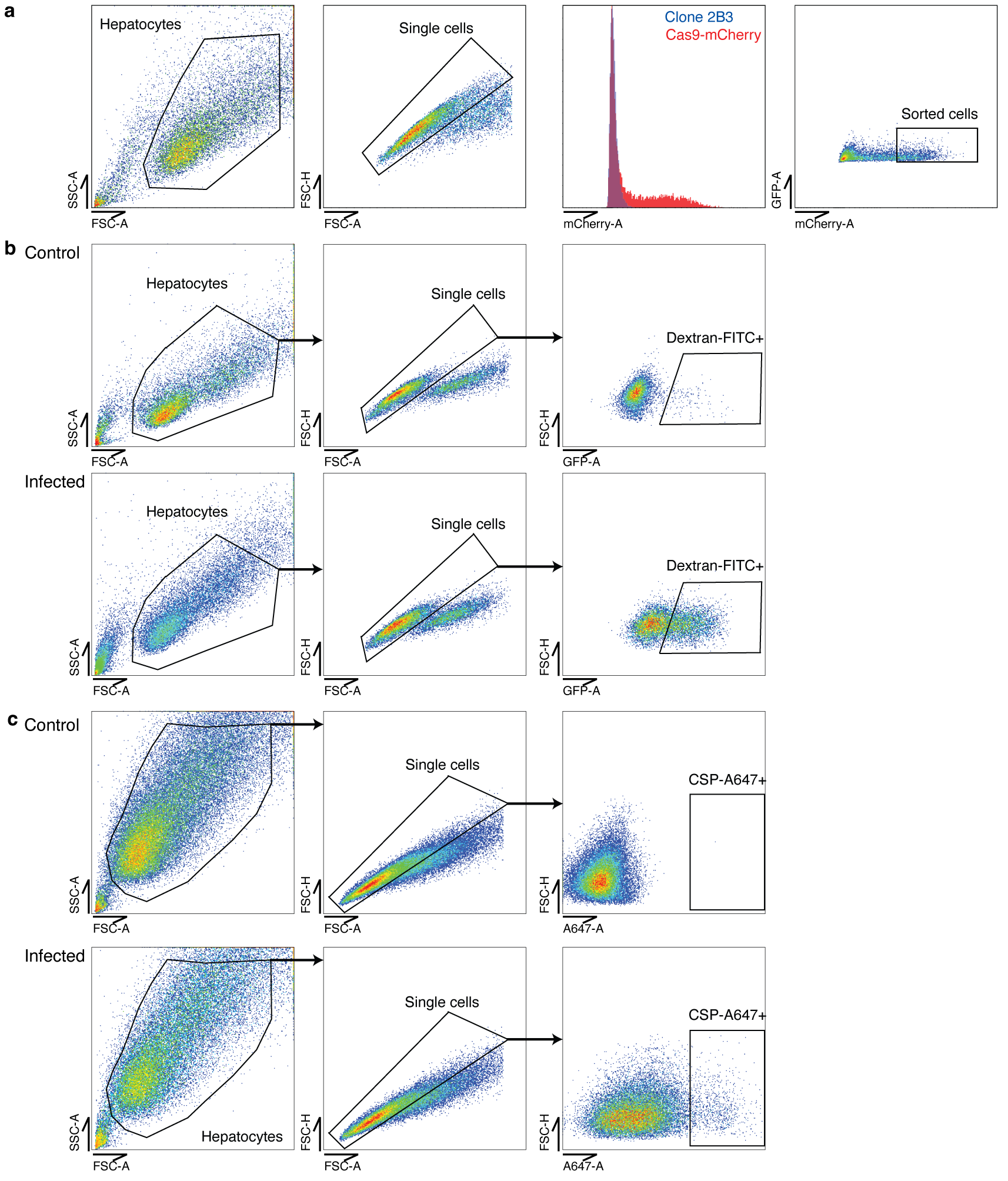

Supplement: S1 Fig — (a) HC-04 cells transduced with Cas9-mCherry construct were analyzed by flow cytometry before FACS cloning. (b) Cell traversal was determined by flow cytometry of live HC-04 4 hours post addition of sporozoites. Cells were gated before singlets were selected and dextran-FITC was determined using a well containing dextran-FITC in the medium but no sporozoites (top). (c) Similar strategy was used to determine invasion, though fixed cells (left) show different scatter profile. (TIF) [file pgen.1012137.s001.tif]
